# Prenatal Anxiety and Exercise. Systematic Review and Meta-Analysis

**DOI:** 10.3390/jcm10235501

**Published:** 2021-11-24

**Authors:** Miguel Sánchez-Polán, Cristina Silva-Jose, Evelia Franco, Taniya S. Nagpal, Javier Gil-Ares, Qin Lili, Rubén Barakat, Ignacio Refoyo

**Affiliations:** 1 AFIPE Research Group, Faculty of Physical Activity and Sport Sciences-INEF, Universidad Politécnica de Madrid, 28040 Madrid, Spain; cristina.silva.jose@upm.es (C.S.-J.); javier.gil@upm.es (J.G.-A.); barakat.ruben@gmail.com (R.B.); 2 Department of Education, Research and Evaluation Methods, Faculty of Social and Human Sciences, Universidad Pontificia de Comillas, 28049 Madrid, Spain; efalvarez@comillas.edu; 3 Department of Kinesiology, Faculty of Applied Health Sciences, Brock University, St. Catharines, ON L2S 3A1, Canada; tnagpal@brocku.ca; 4 Sports and Health Research Center, Department of Physical Education, Tongji University, Shanghai 200092, China; qinlili@tongji.edu.cn; 5 Sports Department, Faculty of Physical Activity and Sport Sciences-INEF, Universidad Politécnica de Madrid, 28040 Madrid, Spain; ignacio.refoyo@upm.es

**Keywords:** physical activity, exercise, pregnancy, prenatal anxiety

## Abstract

The prevalence of prenatal anxiety has increased during the COVID-19 pandemic. Anxiety is associated with other cardiovascular, physiological, and mental illnesses, resulting in adverse health effects for the mother and foetus. The purpose of this study was to evaluate the effects of physical activity (PA) during pregnancy on the prevalence of prenatal anxiety or symptoms of anxiety. A systematic review and two meta-analyses were performed (Registration No. CRD42021275333). Peer-reviewed articles reporting the effect of a PA intervention on anxiety during pregnancy were included. The first meta-analysis (MA) included 10 studies reporting final scores of prenatal anxiety. A negative association between moderate PA during pregnancy and prenatal anxiety was found in this analysis (z = −2.62, *p* < 0.01; ES = −0.46, 95% CI = −0.80, −12, I2 = 84%, Pheterogeneity = 0.001). The second MA included eight studies in which measures both before and after a PA intervention were reported. The findings of this analysis revealed a positive association between exercise practice during pregnancy and a decrease in prenatal anxiety scores (z = −3.39, *p* < 0.001; ES = −0.48, 95% CI = −0.76, −0.20, I2 = 71%, Pheterogeneity = 0.001). Supervised PA during pregnancy could prevent and reduce prenatal anxiety and anxiety symptoms.

## 1. Introduction

For a long time, mental disorders (i.e., depression, anxiety, stress, or dementia) have constituted a worrying problem and their prevalence has increased manifold in recent years. This problem has only grown during the COVID-19 pandemic, increasing the global prevalence of major depression disorder from 24,705 to 31,529 cases per 100,000 population. Additionally, the global prevalence of anxiety disorders has increased from 38,249 to 48,024 cases per 100,000 population. This means an increase of 532 million and 762 million cases of depression and anxiety, respectively, during 2020, with the majority comprising women [1].

Anxiety is a mental disorder characterised by two constructs: state anxiety, directly associated with passing psychological and physiological reactions related to untoward specific situations; and trait anxiety, meaning individually expressed characteristics related with the presence of state anxiety [2]. Nevertheless, trait anxiety scores can be maintained over time in patients with anxiety disorders [3], so anxiety could be determined by one of these constructs because the higher the state anxiety levels, the higher the trait anxiety levels [4,5].

It is not only the general population affected by this illness. Focusing on pregnant populations, mental disorders also pose a great risk to the health of women during pregnancy, even more so when their prevalence has skyrocketed during the COVID-19 pandemic. Approximately 57% of pregnant women during the COVID-19 pandemic presented relevant symptoms of anxiety [6].

Pregnancy could be a vulnerable period for women, especially regarding mental illnesses, which are more likely to manifest during this time compared to other periods of their life [7]. It is known that certain pregnancy risks (such as excessive maternal weight gain) are directly related to an increase in anxiety levels during pregnancy [8]. Prenatal anxiety not only affects psychological parameters, it can also increase the incidence of other pathologies like cancer, heart disease, stroke, arthritis, high blood pressure, and gestational diabetes [9]. The impact of mental disorders even goes beyond the mother’s health. Literature has revealed that the foetus and newborn are not unaffected by the effects of mental disorders. Mothers who experience poor mental health during gestation are more likely to give birth to a low-birth-weight infant and newborns whose mothers have experienced mental disorders during pregnancy are more likely to display worse results on the Apgar test at 1 and 5 min than those whose mothers did not experience mental concerns [10]. The most common treatment for anxiety is the use of antidepressive and anxiolytics drugs. However, most pregnant women do not take them due to the unknown risk that the intake of this medication could have on the foetus’ health [11].

Considering exercise has positive effects on reducing anxiety in the general population and also the fact that exercise has positive effects on other mental disorders in the pregnant population too, it would be logical to postulate that exercise could also be beneficial in prenatal anxiety [12]. It has been shown that physical exercise programs carried out during pregnancy reduce the severity of depressive symptoms and odds of prenatal depression [13].

Due to the publication of recent studies that report the effectiveness of exercise to reduce or prevent depression among pregnant women (e.g., 16% less likely to suffer prenatal depression if the women were active during pregnancy [14]), this systematic review and meta-analysis aimed to summarise the scientific literature to determine the possible effects of physical activity during pregnancy on perinatal anxiety.

## 2. Materials and Methods

Following the Preferred Reporting Items for Systematic reviews (PRISMA) guidelines [15], this study was developed and registered with the International Prospective Register of Systematics reviews (PROSPERO, Registration No. CRD42021275333).

Population, Intervention, Comparison, Outcomes and Study design framework (PICOS) was used to analyse the searching sources included in this research [15]. Articles written in English or Spanish published between 2010 and 2021 were searched.

### 2.1. Population

Pregnant women without any obstetrical relative (e.g., gestational hypertension, malnutrition, or moderate cardiovascular disease) or absolute (e.g., premature labour, pre-eclampsia, or incompetent cervix) contraindications, participating in a physical activity programme during pregnancy were chosen.

### 2.2. Intervention

Intervention characteristics analysed were: (a) weekly frequency of physical activity sessions; (b) intensity: all studies included had a moderate intensity of load, using 55–65% of the maximum maternal heart rate or the perception of effort (range 12–14 of the Borg Scale); (c) duration of the physical activity program; (d) type of physical activity (e.g., Yoga, Pilates, aerobic, strength, or pelvic floor training); (e) supervision or not, of the physical activity program; (f) time duration of the sessions; and (g) if the authors carried out a complementary intervention, as shown in Table 1.

### 2.3. Comparison

Women who engaged in an exercise or physical activity program during pregnancy were compared with those who did not participate in such a program. Intervention characteristics were retrieved and compared as shown in Table 1.

### 2.4. Outcomes

The prenatal anxiety scores (if available, at the beginning and at the end of the intervention) of each questionnaire administered were the target outcome. The following anxiety questionnaires were included in the selected articles: the State-Trait Anxiety Inventory (STAI), Hospital Anxiety and Depression Scale (HADS), and General Health Questionnaire (GHQ-28).

### 2.5. Study Design and Selection Process

The search for this study was done between September and October 2021, at Universidad Politécnica de Madrid (INEF). Medline (PubMed) and SPORTDiscus databases were searched. The search terms were:

- English: physical activity OR exercise OR sport OR fitness AND pregnant population OR pregnancy OR prenatal anxiety OR anxiety OR emotional OR emotional factors OR mental disorders.
- Spanish: actividad física O ejercicio O ejercicio físico O deportes Y población gestante O embarazo O ansiedad prenatal O ansiedad O emocional O factores emocionales O desórdenes mentales.

**Figure 1:**
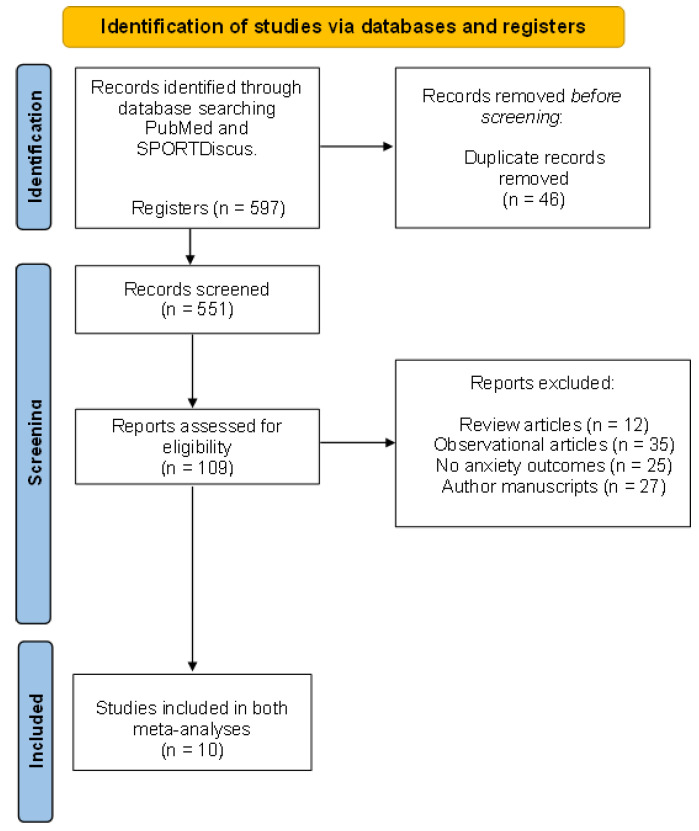
Flow diagram of the analysed articles.

Eligible articles for our review included studies that had to have a quantifiable physical activity or exercise intervention (excluding the articles with only advice to have an active pregnancy or those having a measurable physical activity questionnaire but without an exercise intervention), with prenatal anxiety outcomes (such as state or trait anxiety scores), and with the characteristics of the physical activity or exercise program provided. This process is detailed in Figure 1 [16].

The primary outcome was prenatal anxiety scores assessed by different questionnaires. Studies reporting either trait or state anxiety were included in the review. In the cases in which both trait and state anxiety were reported in the same study, both measures were separately included in the meta-analysis. Along the chosen articles, some studies only reported a final anxiety score (i.e., anxiety was only measured at the end of the intervention program). On the other hand, some studies reported an anxiety score both before and after the intervention program. As it will be described in the next section, a separate meta-analysis was performed for each of these studies to better reflect the existing evidence. Additionally, to check the effects of each intervention on maternal health, other outcomes were examined (but not included in the meta-analyses) as secondary outcomes like physiologic, sociodemographic, and delivery outcomes.

From each chosen study, we extracted: author(s), publication year, country in which the study was developed and type of study design, number of participants, characteristics of the intervention program, variables analysed (primary and secondary), and if any type of cointervention was included (Table 1).

**Table 1:** Characteristics of the studies analysed.

Author, Year and Country	N; IG; CG	Intervention. Physical Activity Program						Main Variables Analysed	Secondary Variables Analysed	Co-Intervention
		W Freq.	Int.	Time	Type	Sup.	Duration			
Rong et al., 2021. China [17]	64; 32; 32	3	Mod	22–34 *	Yoga	Yes	60 min	Physiological discomforts, prenatal depression and anxiety	Childbirth self-efficacy and delivery outcomes	No
Gallagher et al., 2020. United States [18]	79; 48; 31	2	Mod	2–32 *	Yoga	Yes	30 min	Prenatal anxiety and depression	Demographic data	No
Mohyadin et al., 2020. Iran [19]	84; 42; 42	3	Mod	26–36	Yoga	Yes	20–60 min	Prenatal anxiety, labour pain, and length of labour stages	Neonatal apgar score, mode of delivery, and demographic data	No
Davis et al., 2015. United States [20]	46; 23; 23	1	Mod	20–28 *	Yoga	Yes	75 min	Prenatal anxiety and depression	Maternal outcomes	No
Guszkowska et al., 2015. Poland [21]	109; 62; 47	2	-	27–35 *	Pilates, Yoga, body ball, muscle strength, stretching and joint mobility exercises	Yes	50 min	Mental health (somatic symptoms, anxiety and insomnia, social dysfunctions severe depression	Sociodemographic variables	Traditional prenatal classes
Field et al., 2013. United States [22]	79; 40; 39	1	Mod	22–34	Yoga	No	20 min	Prenatal and postnatal anxiety and depression	Different hormone levels	No
Field et al., 2013. United States [23]	75; 37; 38	1	Mod	22–34 *	Tai-chi/Yoga	Yes	20 min	Prenatal anxiety and depression	Psychotic and somatic disorders in pregnancy	No
Satyapriya et al., 2013. India [24]	96; 51; 45	7	Mod	18/20–34/	Yoga	Yes	60 min	Prenatal anxiety and depression	Sociodemographic data	Yoga sessions at home
Field et al., 2012. United States [25]	84; 28; 28–28	2	Mod	20–32 *	Yoga	Yes	20 min	Prenatal anxiety and depression	Back and legs pain	No
Ji et al., 2010. Korea [26]	70; 33; 37	2	Mod	23–35 *	Qi exercises (yoga, breathing and meditation exercises)	Yes	90 min	Maternal/foetal interaction, prenatal depression and anxiety, and physical well-being	Sociodemographic data	No

IG, intervention group; CG, control group; W freq., weekly frequency; Int., intensity; Mod, moderate; Time, gestational weeks between intervention was developed; Sup., supervised sessions. * Authors provided mean gestational age at the beginning, and weeks of intervention duration.

### 2.6. Statistical Analysis, Quality of Evidence Assessment, and Risk of Bias

As mentioned earlier, two separate meta-analyses were performed. These analyses were calculated using the overall confidence interval (CI), calculated for both using the standardised mean difference [27].

The first meta-analysis included the studies that only reported anxiety scores at the end of the intervention. On the other hand, the second meta-analysis included studies in which anxiety was measured both before and after the intervention.

In both analyses, each study had a weight relative assignment regarding its sample size number, which contributed to the entire analysis, establishing the compensated average. The I^2^ statistic was used to quantify the heterogeneity present in the results due to the different interventions and designs of each article, indicating the variability of the effect of each intervention, and was not random. The following considerations were used: low heterogeneity = 25%; moderate heterogeneity = 50%; and high heterogeneity = 75% [28].

Ferreira-González et al. [29] demonstrated that, in the case of high heterogeneity, one solution could be to divide the studies into subgroups performed with different characteristics explaining this variability. However, for our article with limited results, we understood that presenting all articles in each analysis would provide a better approach of the study.

For assessment of the quality of evidence for the main outcome and each study, the Grading of Recommendations Assessment, Development and Evaluation (GRADE) framework was used, including studies rated as having moderate or high quality [30]. To determine the potential risk of bias (with these sources: selection, performance, attrition, detection, and reporting bias), the Cochrane Handbook was followed [31]. Randomised clinical trials’ evidence initially started with a “low” risk of bias (due to the theoretical study design and intervention), compared to non-randomised interventions, and both increased or decreased its risk of bias in function of having any “high” or “low” score across bias sources.

## 3. Results

In total, 597 articles were retrieved in the first stage of the search, 46 of which were removed as they were identical to other selected articles and 442 of which were excluded because they did not meet the inclusion criteria (Figure 1). After, 99 articles were excluded since they did not report a quantifiable exercise (or physical activity) intervention, did not report anxiety scores’ standard deviation (SD), or reported inconsistent results according to the questionnaires used in the study. Finally, 10 studies (n = 786 women) were included for analysis.

**Figure 2:**
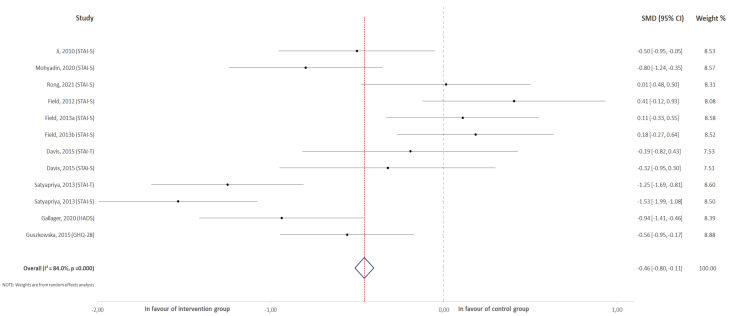
Effect of physical activity on the obtained prenatal anxiety score.

**Figure 3:**
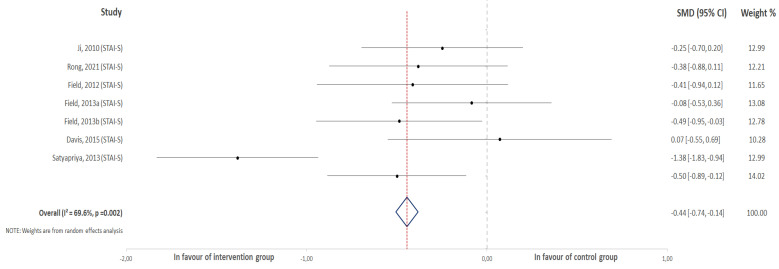
Effect of physical activity during pregnancy on the score change for overall anxiety.

For the first meta-analysis, all the selected studies were included that reported anxiety scores (both trait and state anxiety) that measured this outcome at the end of the intervention (Figure 2). On the other hand, eight of them were represented in the second meta-analysis (Figure 3). In this group, studies exclusively reported state anxiety both at the beginning and at the end of the intervention.

As for the type of intervention reported in the included studies, nine of them described physical activity sessions conducted by professionals in the field. Sessions among chosen studies were designed for moderate intensity and performed with a frequency of one to seven days per week, with a time duration of 20 to 90 min. Duration of each intervention oscillated between 2 and 16 weeks.

### 3.1. Effect of Physical Activity on the Overall Score for Anxiety

Ten different studies were included in this analysis, comparing scores after an intervention for anxiety between experimental and control groups. Two articles [20,24] reported both state and trait anxiety, and these results were included in this analysis. The results revealed a negative association between exercise practice during pregnancy and the scores obtained for the questionnaires that were used to measure anxiety in pregnant women (z = −2.62, *p* < 0.01; ES = −0.46, 95% CI = −0.80, −12, I2 = 84%, Pheterogeneity = 0.001). Figure 2 shows the forest plot corresponding to the present meta-analysis.

### 3.2. Effect of Physical Activity on the Score Change for Overall Anxiety

Eight studies were included in this analysis comparing the difference between the scores after and before the intervention in the experimental and control groups. The results revealed a positive association between physical activity practice during pregnancy and a decrease in the scores obtained for the questionnaires that were used to measure anxiety in pregnant women (z = −3.39, *p* < 0.001; ES = −0.48, 95% CI = −0.76, −0.20, I2 = 71%, Pheterogeneity = 0.001). Figure 3 shows the forest plot corresponding to the present meta-analysis.

### 3.3. Risk of Bias Assessment

**Figure 4:**
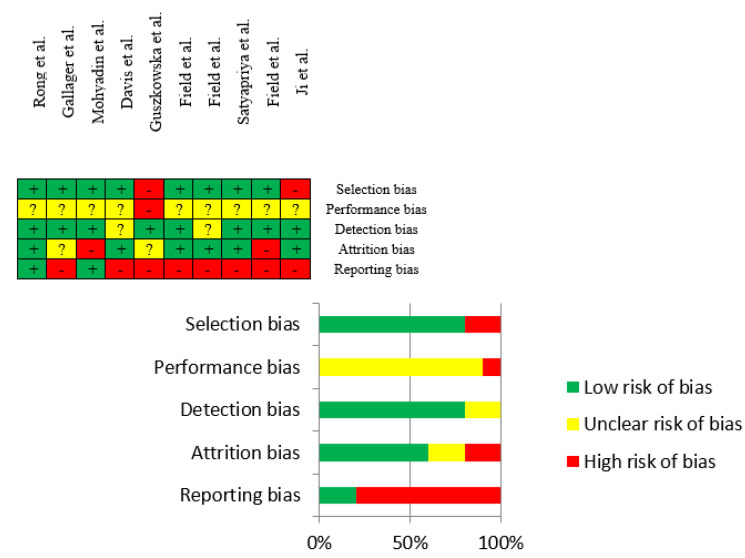
Risk of bias summary from the retrieved articles.

Overall, the risk of bias of each article was rated as low, unclear, or high potential risk (Figure 4). Reviewing sources of bias, the articles showed mostly a low risk of bias on selection, detection, and attrition bias. The majority of the studies presented an unclear performance risk of bias because the blinding of participants in this type of intervention (with a physical exercise group and a control care group) is practically impossible. Additionally, high reporting risk of bias was common across the selected articles due to the inaccessibility of each protocol or that there were some differences between the initial outcomes (written in the protocol) and final measured outcomes in the article.

Even so, and considering the scarcity of studies on this topic, we decided not to discard the articles with an unclear or high risk of bias. Studies showed greater low risk of bias (in general). Although the high unclear percentage of risk of bias findings in the performance source, we followed Cochrane’s Tool considering the difficulty in these types of studies when it comes to blind participants [31]. Despite the high risk of bias scored in the reporting source, the outcomes of interest of each article (even the inaccessibility of their protocols) could not be related to anxiety or anxious symptoms.

## 4. Discussion

This work aimed to determine the effects of physical activity across prenatal anxiety or anxiety symptoms during pregnancy. The present study findings revealed a positive effect of physical activity programs on the reduction of risk of prenatal anxiety. More specifically, it was found that prenatal anxiety symptoms were presented to a greater extent in those women who remained inactive during pregnancy (meta-analysis 1) and physical activity could reduce anxiety scores compared to usual care interventions (meta-analysis 2).

Our findings led us to determine the importance of prescribing physical activity during pregnancy as a tool to prevent and reduce prenatal anxiety. It is known that pregnancy is a difficult period (emotionally speaking) in which women could experience emotional ups and downs manifesting since the first trimester; so, the best intervention approach would begin as soon as possible, and last to delivery if possible. Interventions analysed lasted between 2 and 16 weeks of duration. The study developed by Satyapriya et al. [24] used a duration of 16 weeks, finding that the state and trait anxiety scores were lower in the intervention than the control group and confirming the positive effects of supervised physical activity during pregnancy.

Additionally, these positive effects were demonstrated in several articles in which supervised physical activity interventions have shown a reduction of prenatal anxiety scores in intervention groups compared to control groups [18,19,20,21,24,26]. All the studies included a yoga intervention (or yoga exercises combined with other exercises), with stretching, muscle strengthening, pelvic floor exercises, coordination, balance, and flexibility exercises.

Another reported characteristic of the intervention was the time duration of the sessions performed. We would like to draw attention to the studies with poor anxiety results in the intervention group [22,23,25], which used a shorter time duration (20 min) and lower weekly frequency (1 to 2 days per week) of physical activity sessions.

It is interesting to see that yoga was the most common intervention across the chosen articles. The fact that most interventions consisted of group physical activity sessions could lead us to suggest a beneficial role of socialization that occurred during the program. It would be interesting for future studies to explore how physical activities differ from other group leisure-time activities regarding their effect on mental health during pregnancy. These good results may also be due to the group character of the sessions.

Due to the similarity of symptoms, it is possible to speculate that the reduction in prenatal depression symptoms observed after exercise interventions reported in recent systematic reviews [14,32] could be comparable and transferable to prenatal anxiety outcomes. This idea is demonstrated in other systematic reviews, showing better results in the intervention than in the control groups [33,34].

### Strengths and Limitations

The limitations of this study were the shortage of published articles that measured prenatal anxiety, performed a quantifiable intervention (different than the program followed in the control group), and used measures of anxiety during and not after the pregnancy. Due to the limited number of selected papers, we decided to include articles with different questionnaires administered during pregnancy. Although the powerful relationship between having high scores in anxiety questionnaires and diagnosed anxiety, it is worth mentioning that in the majority of the reviewed articles, anxiety was not diagnosed (even so, the questionnaires offer cut-off points to assume that the woman has prenatal anxiety or symptoms). To demonstrate these positive results, a randomised clinical trial will be developed.

## 5. Conclusions

Supervised physical activity during pregnancy could be a good approach to prevent and reduce prenatal anxiety and anxiety symptoms.

## Data Availability

The data presented in this study are available on request from the corresponding author.
